# Stroke in children and adolescents: Analysis of electrophysiological and behavioral assessment findings of auditory processing

**DOI:** 10.1016/j.clinsp.2023.100286

**Published:** 2023-10-07

**Authors:** Andréia Rodrigues Parnoff Stadulni, Pricila Sleifer, Amanda Zanatta Berticelli, Rudimar Riesgo, Carolina Nunes Rocha-Muniz, Eliane Schochat

**Affiliations:** a Department of Physiotherapy, Speech Therapy and Occupational Therapy, Faculdade de Medicina da Universidade de São Paulo (USP), São Paulo, SP, Brazil; b Department of Health and Human Communication, Universidade Federal do Rio Grande do Sul (UFRGS), Porto Alegre, RS, Brazil; c Graduate Program in Child and Adolescent Health, Faculdade de Medicina da Universidade Federal do Rio Grande do Sul (UFRGS), Porto Alegre, RS, Brazil; d Hospital de Clínicas (HCPA), Porto Alegre, RS, Brazil

**Keywords:** Children, Adolescents, Stroke, Hearing, Auditory evoked potentials, Auditory processing disorders

## Abstract

**Purpose:**

This study aimed to analyze the auditory processing behavior of children and adolescents diagnosed with stroke and compare it with that of typically developing individuals.

**Methods:**

This was an analytical cross-sectional study involving 48 participants aged between 7 and 17 years with adequate schooling for age and grade, allocated equally to two groups: Stroke (SG) and Control Groups (CG). For the SG, cases identified between 2003 and 2018 were considered. In the CG, school-aged participants with typical development were randomized. After screening for differential audiological assessment and confirmation of auditory pathway integrity at the brainstem level, binaural analyses of the auditory processing behavior were conducted using the Dichotic Digit Test (DDT), Frequency Pattern Test (FPT), and electrophysiological assessment (P300). The Shapiro-Wilk test for normality was conducted, followed by the T and Mann-Whitney tests, with a 95 % confidence level and significance offset at *p* < 0.05, using the SPSS software (IBM®, v. 22.)

**Results:**

The CG performed better in terms of auditory processing. These differences were significant (*p* < 0.0001) for the binaural integration of DDT, FPT humming and Labeling, and P300 latency. The P300 results were similar; however, with a greater amplitude in the SG.

**Conclusion:**

This study showed that children and adolescents with stroke performed worse in electrophysiological and behavioral tests of auditory processing assessed using the auditory evoked potentials. These data reinforce the hypothesis that stroke-related lesions compromise the neural mechanisms underlying auditory processing.

## Highlights

• Auditory processing behavior of children diagnosed with stroke was analyzed.
• Children and adolescents with stroke performed worse in auditory processing tests.
• Stroke-related lesions compromise the auditory neural mechanisms.

## Introduction

Cerebral Vascular Accidents (CVA) in children and adolescents are rarely described. However, they are becoming increasingly important, and are emerging conditions in the field of research because of complications and require high diagnostic accuracy, as the signs and symptoms manifested initially may have little specificity, with clinical presentations being similar to those of other neurological diseases or those related to the central nervous system.^1^^,^^2^

Most available studies on pediatric stroke have small sample sizes, making the diagnostic approach, treatment, and determination of prevalence rates difficult.^3^ In this context, the literature review has made important advances in identifying the etiology of the disease. The most common causes are vascular malformations, followed by Moyamoya Syndrome, arterial dissection, heart disease and cardioembolic phenomena, sickle cell anemia, adverse effects of other treatments, brain tumors, and trauma;^3^, ^4^, ^5^ and more than 27 % of cases may have an unknown etiology.^3^

Stroke can occur due to sudden occlusion, causing ischemia or infarction in the encephalic territory, and is characterized as an acute ischemic stroke. It can also present in a hemorrhagic form when the cerebral veins or arteries rupture, and both forms can result in focal lesions and neurological clinical deficits.^6^ The international incidence rates range from 1.2 to 13 per 100,000 children.^5^, ^6^, ^7^, ^8^, ^9^, ^10^

Due to the challenges involved in early diagnosis, it is possible that the incidence of pediatric stroke is underestimated. In contrast to hemorrhagic stroke in adults, which is present in 7.5–19 % of cases, hemorrhagic events in children are present in 35–54 % of cases.^5^ The disease in this population has a devastating effect on the quality of life and has a significant socioeconomic impact. Deaths occur in up to 10 % of cases with recurrence in 20 %, and 75 % of children remain with neurological problems such as seizures, sensory and motor deficits, behavioral disorders, and intellectual disability, which hinder learning and language.^11^

Another aggravating aspect is that childhood strokes can affect all levels of the auditory pathway, leading to deficits in reception and/or auditory perception. These can manifest as a variety of symptoms and clinical presentations that begin acutely before, during, or shortly after the stroke.^12^ For this population, the integrity of the auditory pathway and the typical development of the neural mechanisms underlying auditory processing are fundamental for the acquisition and development of language and learning processes.^13^

According to the American Speech-Language-Hearing Association (2005) and the American Academy of Audiology (2010), postnatal events such as trauma or neurological infection can result in an acquired auditory processing disorder.^14^^,^^15^ Neurodevelopmental and auditory processing disorders after stroke have not been frequently studied and are still poorly documented, despite the relevance of this topic.^16^ A systematic review published in 2023 investigated the interventions applied to perception disorders in children after stroke. Randomized clinical trials reported touch disorders, mixed tactile-somatosensory and somatosensory disorders, and changes in visual perception. However, studies that addressed hearing, which depends on the specific mental functions of recognition and interpretation remain lacking in the literature.^17^

Considering the rarity of this clinical condition and the importance of investigating auditory skills, the aim of this study was to analyze the findings of electrophysiological assessments and auditory processing behavior of children and adolescents diagnosed with stroke and compare them with healthy individuals with typical development.

## Material and methods

### Design

This analytical cross-sectional study was approved by the Research Ethics Committee under protocol nº 77900517.2.0000.5334 and was conducted in accordance with the Declaration of Helsinki and STROBE guidelines. This study was conducted at the Center for Auditory Electrophysiology of the Federal University of Rio Grande do Sul (UFRGS) and the Department of Physiotherapy, Speech Therapy, and Occupational Therapy of the University of São Paulo (USP) School of Medicine, São Paulo (SP). Free and informed consent was obtained from all participants and their respective parents.

### Sample

The study analyzed children and adolescents who were included after their parents provided informed consent. The samples were then divided into two groups.

Stroke Group (SG): Children and adolescents diagnosed with stroke were treated and followed up at a neuropediatric reference unit in Porto Alegre, Rio Grande do Sul, Brazil.

Control Group (CG): Healthy children and adolescents with typical development were recruited from public schools in the same city.

To make up the SG, the cases identified during the period were considered, while for the CG, individuals were randomized from a sample of 500 school-age children and adolescents with typical development for data comparison purposes.

### Inclusion and exclusion criteria

The following were considered for both the study groups: age between 7 and 17 years, 11 months, and 30 days; minimum follow-up time of 1 year after stroke; appropriate school performance for age and school year; no previous auditory complaint; adequate auditory capacity with a type A tympanometric curve; and integrity of the auditory pathways at the level of the brainstem, as confirmed by screening and neuropediatric evaluation of the participants.

Patients with SG were diagnosed with ischemic or hemorrhagic stroke between 2003 and 2018. In the CG, school-aged children and adolescents selected and assessed between 2016 and 2018 were included.

Individuals in both groups with neurological sequelae that precluded audiological assessment were excluded.

### Screening

All participants underwent screening and binaural assessment by an audiologist using the following institutional protocol:

I Anamnesis and overall data collection as well as medical history and ontological data.
II Meatoscopy to determine the presence of cerumen or foreign bodies.
III Immittance testing with an Interacoustics® AT235h Impedance Audiometer, in order to identify the tympanometric curve and analyze ipsilateral and contralateral acoustic reflexes at frequencies of 500, 1000, 2000, and 4000 Hz.
IV Pure-tone audiometry, which followed the American National Standards Institute (ANSI-69) guidelines, assessed the auditory thresholds at frequencies between 250 and 8000 Hz.^18^
V Vocal audiometry was used to determine the Speech Percentage Recognition Index (SPRI) and Speech Recognition Threshold (SRT), followed by the presentation of twenty-five monosyllabic words at 40 dB HL, the intensity of which gradually declined until the participant understood and repeated 50 % of the words.
VI Behavioral and electrophysiological assessments were performed using the Auditory Brainstem Response (ABR), which consists of emitting specific auditory evoked potentials at 80 dB HL with alternate polarity, to confirm the auditory pathway integrity at the brainstem level.^19^

The screening data were used to select the study participants whose data were not used to demonstrate the results.

### Behavioral analysis of auditory processing

The study analyzed the participants’ central auditory processing using the Dichotic Digit Test (DDT), Frequency Pattern Test (FPT), and electrophysiological assessment (P300).

The DDT is a behavioral test of central auditory processing that assesses the perception and understanding of speech sounds related to the acquisition and understanding of language. In addition to the auditory system, the test challenges cognitive functions as it requires attention and memory for execution.^20^^,^^21^ This evaluation method is useful for screening auditory processing disorders and stands out for its low complexity and practicality of application, thus enabling its use for screening in a school environment. It is also recommended for the evaluation of individuals with neurological sequelae, with a sensitivity and specificity of 90 % and 83 %, respectively.^21^

The procedures described in the auditory processing evaluation manual were adopted for this application.^22^ A recording was turned on the acoustic booth, with an intensity of 50 Db HL in relation to the SRT, which repeated a list with 25 sequences of digits presented in a dichotic and random manner (e.g., three, five, six, and eight), totaling 100 digits in each ear. The number of correct answers was converted into a percentage to be analyzed later. The test evaluates the binaural integration based on the participant's ability to repeat the presented numbers orally. The binaural separation was determined by the ability to repeat the numbers presented orally to each ear.

The FPT is used to assess the auditory electrophysiological potential involved in central auditory processing and is characterized by its ability to determine the location and lateralization as well as the recognition and discrimination of sound characteristics in environments with competitive noise.

This test was also conducted in an acoustic booth, using 40 sequences of sounds recorded at 50 dB HL above the tritonal average, at frequencies between 500 and 2000 Hz. Three different acoustic signals were presented in each sequence, of which two sounds were emitted at the same frequency and the third at different frequencies. Each tone was parameterized with duration, interval, and rise and fall times of 200, 150, and 10 ms, respectively. The evaluation took place in two stages, the first instructing the participant to imitate the sound (humming) and the second to characterize the sounds in categories “high-pitched or shrill” or “coarse or deep” (labeling). The answers were converted into a percentage of correct answers, considering ≥75 % as the standard of normality.^23^

For the P300, four electrodes were placed: one on the forehead, one on the scalp, and two on the mastoid processes. The impedance was regulated until reaching intensities ≥ 5 Ω, and a difference ≥ 2 Ω between electrodes was accepted. Subsequently, an Electroencephalography (EEG) was performed to record spontaneous brain activity and identify artifacts, followed by an ABR test.

After this preliminary stage, P300 was applied using a Contronic® Masbe ATC Plus device, and the participants were instructed to speak or wave their hands whenever they perceived a different sound signal. The binaural test exhibited a sequence of frequent stimuli (tone burst at 1000 Hz), and different and less frequent stimuli presented randomly (tone burst at 2000 Hz). The following criteria were used: 200 µV full scale; 20 ms plateau and rise and fall times of 5 ms; 0.5 Hz high-pass filter; 20 Hz low-pass filter, Notch – SIM; 1000 ms reading window; presentation of 300 stimuli, 20 % of which were rare. The P300 wave latency was marked at the point of maximum amplitude, and all electrophysiological recordings were analyzed by two experienced examiners. The latency data were recorded in milliseconds (ms) and amplitudes in microvolts (µV).^24^

In addition to the data obtained from the auditory processing assessment, the following data were collected: stroke classification, age at stroke onset, age at assessment, sex distribution, and the brain region affected.

### Statistical analysis

The Shapiro-Wilk test was conducted to determine the data normality in each auditory processing assessment, followed by non-parametric tests to ascertain the differences between the study groups.

The *t*-test was applied to compare the normally distributed data with those obtained using the Mann-Whitney *U* test for analyses that used non-normally distributed data. A 95 % Confidence Interval was established at a significance level of *p* < 0.05. The tests were performed using the Statistical Package for the Social Sciences (SPSS) software version 22.

## Results

Thirty-five children/adolescents diagnosed with stroke between 2003 and 2018 were identified, 25 of whom agreed to participate in the study and 24 of whom met the inclusion criteria (SG). In addition, 24 individuals with typical development (CG) were included. Therefore, this study included 48 participants.

The entire sample suffered a stroke between 2 and 10 years of age, and it was found that in 91.67 % of the cases, the stroke occurred in early childhood (before four years of age). All participants were evaluated in the chronic phase after stroke, with the shortest follow-up time recorded at the time of the assessments being 1 year and the longest being 12 years after stroke. Most patients were girls, and the stroke was ischemic in the left cerebral hemisphere. The epidemiological profiles of the study participants are presented in Table 1.

**Table 1:** Descriptive analysis of the epidemiological profile of participants in the experimental and control groups regarding age, sex, brain region, and type of stroke.

Variables		SG	% (SG)	CG	%(CG)
Age	Stroke	3.84 ± 1.82		‒	‒
	Evaluation	11.40 ± 2.57		10.48 ± 2.08	‒
Sex	Female	13	54.17	13	54.17
	Male	11	45.83	11	45.83
Brain Stroke	Ischemic	22	91.67	‒	‒
	Hemorrhagic	2	8.33	‒	‒
Brain region	Right hemisphere	6	25	‒	‒
	Left hemisphere	15	62.5	‒	‒
	Cerebellum	2	8.33	‒	‒
	Brainstem	1	4.17	‒	‒

Significant differences in auditory processing were observed between children diagnosed with stroke and those with normal development.

With respect to DDT, the means and standard deviations for the “integration” variable analyzed in the right ear was SG: 76.83 ± 16.35 and CG: 99.90 ± 0.50, and for the left ear were SG: 82.67 ± 15.48 and CG: 99.00 ± 1.25. A comparative intergroup analysis revealed a statistically significant difference in integration capacity (*p* < 0.0001).

**Fig. 1:**
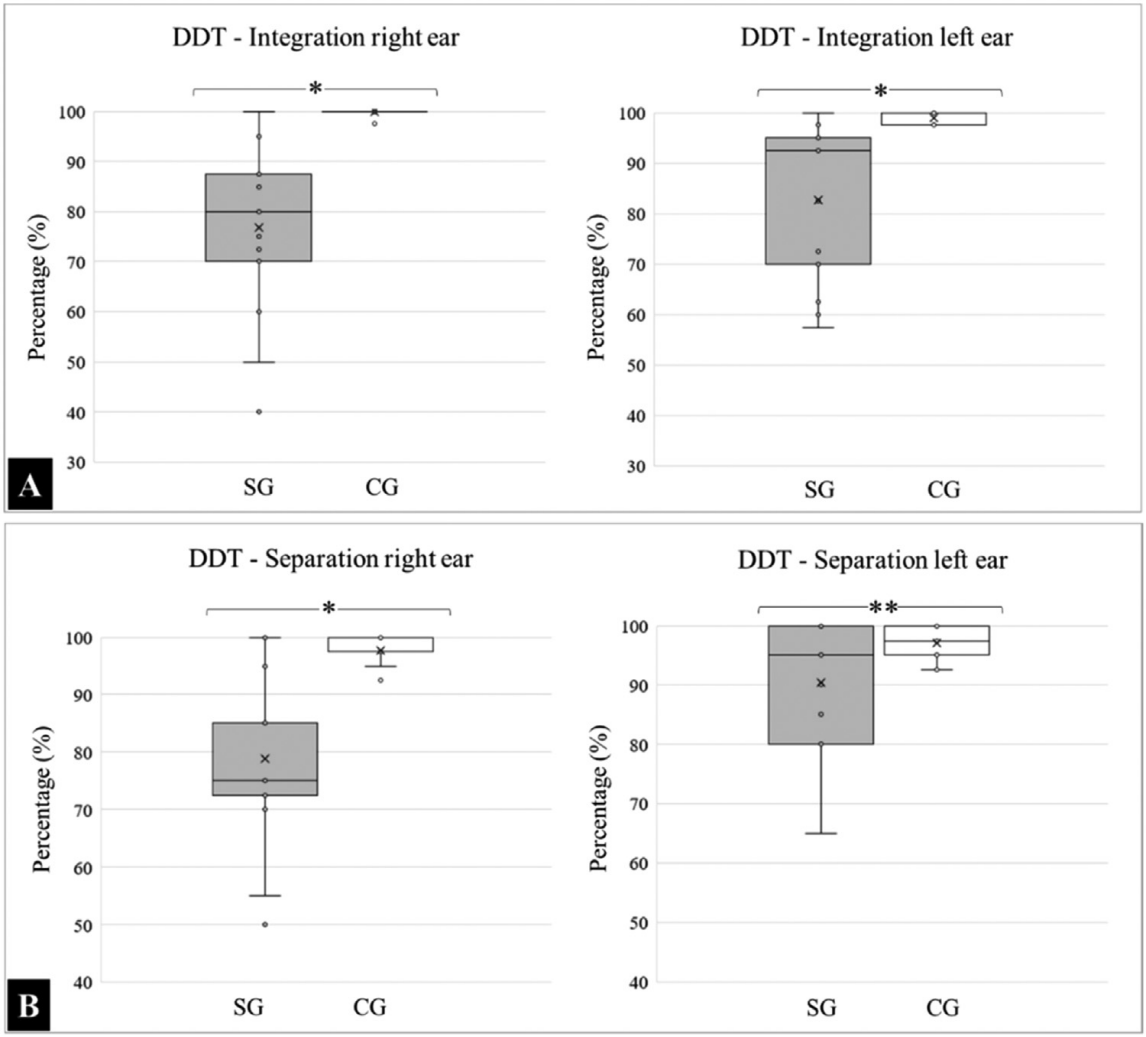
Percentage of correct answers for the integration (A) and separation (B) variables identified by DDT for the SG and CG. (*) Significant statistical difference, *p* < 0.0001; (**) Statistically similar data.

For the “separation” variable, the results for the right ear were SG: 78.83 ± 14.36 and CG: 97.70±2.69, and for the left were SG: 90.33 ± 10.43 and CG: 97.10 ± 2.47. Regarding separation, there was a significant difference in the right ear (*p* < 0.0001); however, the findings in the left ear were statistically similar (*p* = 0.0716). The medians, quartiles, and outliers for each variable are presented in Fig. 1.

**Fig. 2:**
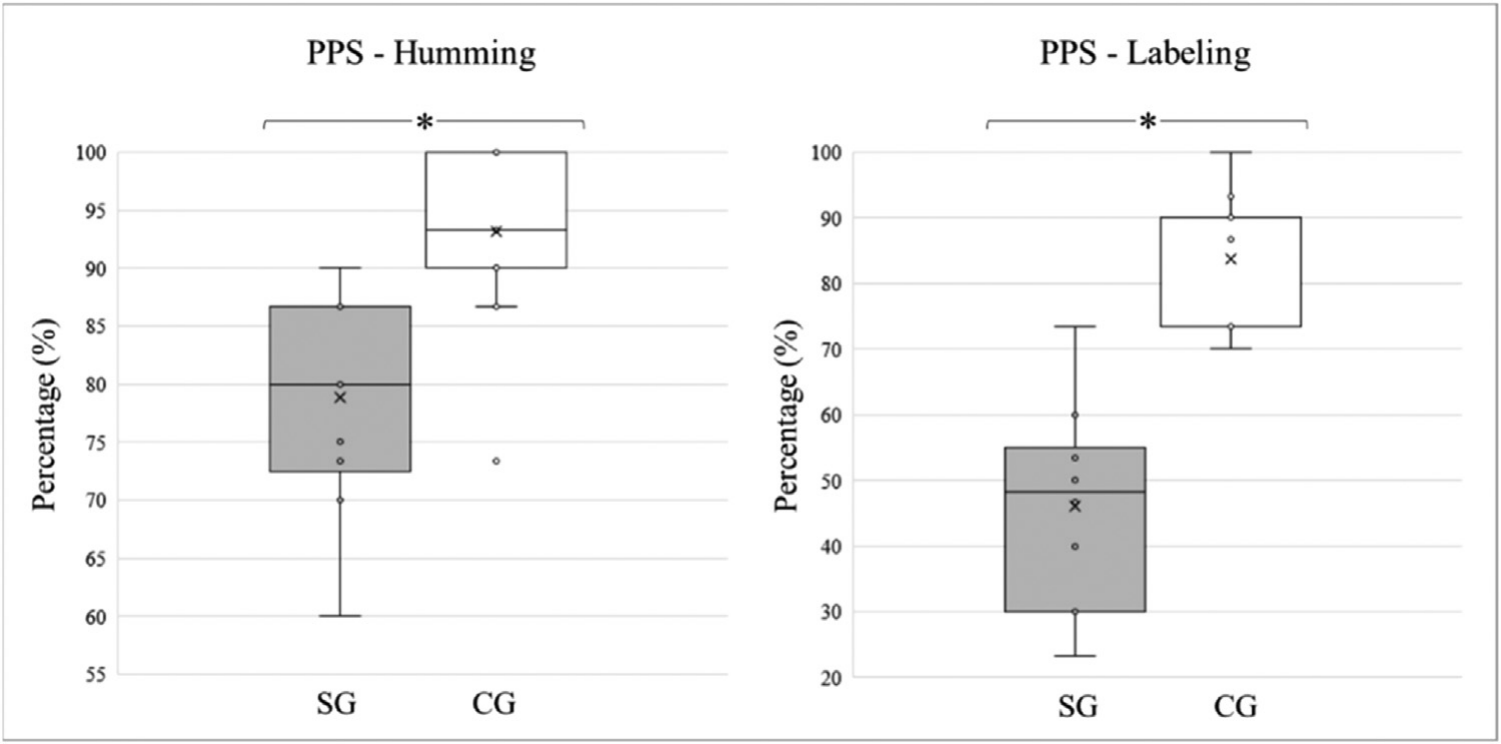
Percentage of correct answers for PPS Humming and Labeling modalities for the SG and CG. * Significantly different (*p* < 0.0001).

The students with typical development performed better on the FPT, obtaining a higher percentage of correct answers when compared to the children and adolescents diagnosed with stroke, in both “labeling” and “humming”. The average “humming” percentages and standard deviations for the SG and CG groups were 78.83 ± 9.36 and 93.20 ± 6.49, respectively. For “labeling,” the percentage of correct answers was 46.00 ± 15.38 for the experimental group and 83.73 ± 9.30 for controls. The differences between the modalities were statistically significant (*p* < 0.0001). The medians, quartiles, and outliers for each variable and study group are shown in Fig. 2.

Children and adolescents with typical development also performed better in the electrophysiological assessment of the P300 test, and the differences were significant for latency and similar for binaural response amplitude.

The mean and standard deviations of the latency, expressed in milliseconds (ms) in the right ear, for the SG and CG, were 495.96 ± 112.91 and 306.43 ± 11.90, respectively; (*p* < 0.0001), and 502.84 ± 113.18 and 306.96 ± 18.34, respectively for the left ear (*p* < 0.0001).

**Fig. 3:**
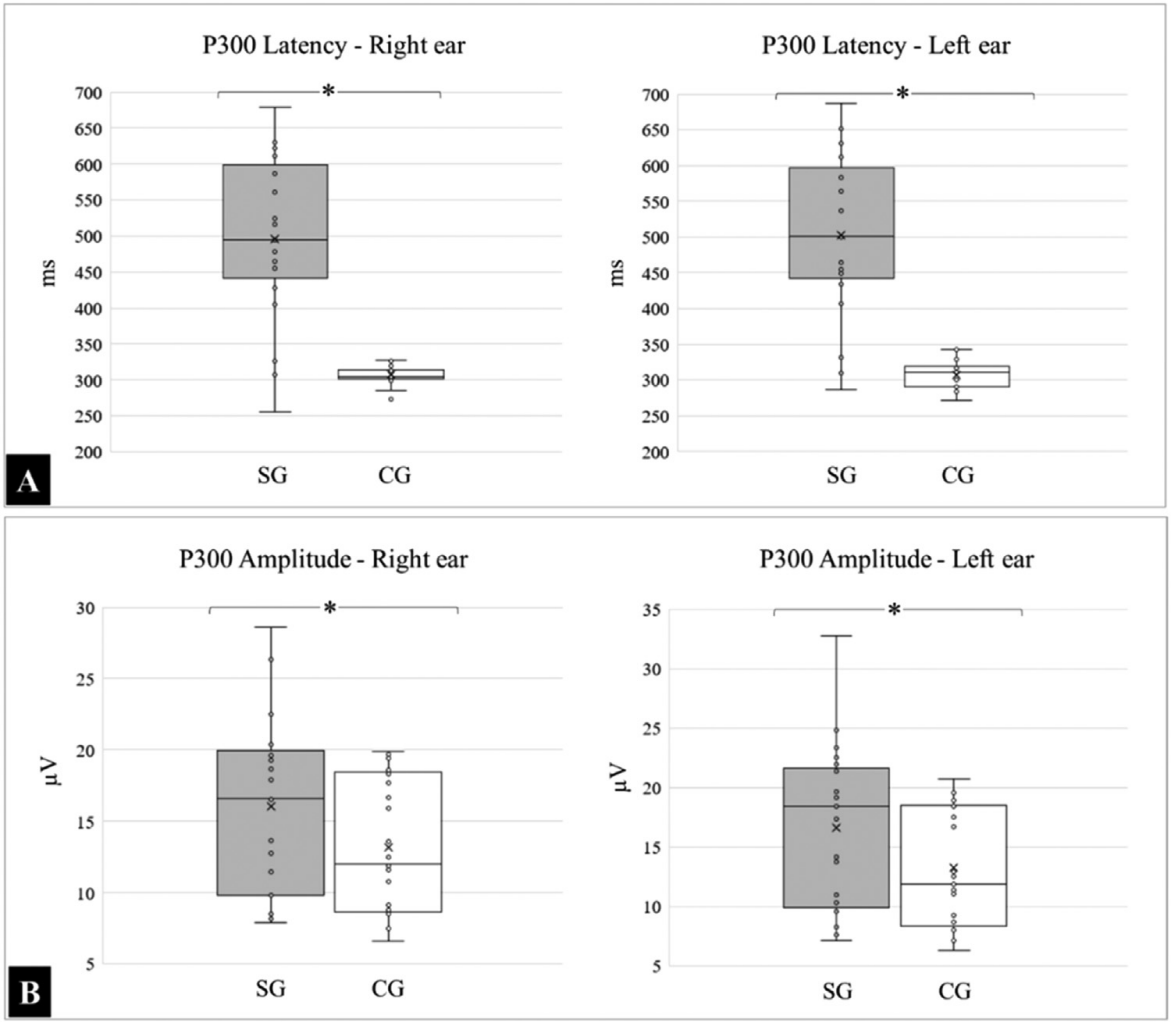
Latency (A) and amplitude (B) measured during P300 in groups SG and CG. (*) Significant statistical difference, *p* < 0.0001.

For the amplitudes, expressed in microvolts (µV), the following means were obtained in the right ear for the SG and CG: 16.07 ± 6.18 and 13.15 ± 4.78 respectively; (*p* = 0.0777), and 16.63 ± 6.83 and 13.22 ± 4.95, respectively for the left ear; (*p* = 0.0566). The medians, quartiles, and outliers for latency and amplitude in both groups are shown in Fig. 3.

## Discussion

Stroke has a significant functional impact on an individual's quality of life.^25^ However, few studies have investigated the impact of stroke on central auditory pathways in the brain. The authors believe that this scarcity is because other morbidities resulting from stroke could ‘mask’ the difficulties in auditory processing skills. Patients typically report hearing difficulties only when asked about or evaluated using specific tests.^12^^,^^26^^,^^27^

The present study described the function of the auditory pathway in children and adolescents with stroke and proposed new therapeutic perspectives that can help recover communicative functions in this population.

The present data demonstrated a close connection between the structural damage caused by stroke and impaired auditory pathway functionality (auditory perception skills), both through behavioral and electrophysiological tests.

The SG comprised 24 individuals: 13 girls (54 %) and 11 boys (46 %). Some studies have reported a higher prevalence in males^7^^,^^25^^,^^28^, ^29^, ^30^, ^31^ than in females.^32^, ^33^, ^34^, ^35^, ^36^

Regarding the type of stroke, most of the sample consisted of ischemic stroke (92 %). This prevalence has been corroborated by several studies on stroke in both adult and pediatric populations.^30^^,^^34^^,^^36^ This is because most of the published studies were conducted in follow-up and rehabilitation centers, and in cases of hemorrhagic stroke in children, the number of deaths was higher.^25^^,^^34^^,^^37^

The injury locations in the study population showed a wide diversity in neuroimaging findings. However, most patients had an injury to the temporal left hemisphere (63 %). This is due to the location of the Middle Cerebral Artery (MCA), the most commonly affected vascular region, which is more prone to injury due to its anatomy and thinner walls.^34^^,^^36^^,^^38^

The results of the behavioral tests, DDT, and PPS, showed that the SG performed worse than the CG in all the tasks.

In the DDT, the SG performed worse in the tasks of integration and binaural separation in both ears than the CG. These findings corroborate those of other studies that examined the same population.^29^^,^^39^

In the dichotic listening task, different verbal stimuli were presented concurrently to each ear. To evaluate the binaural integration, the authors measured the neural capacity to integrate each stimulus presented simultaneously to both ears. With respect to binaural separation, the authors evaluated the neural capacity to direct attention to each ear. That is, information from one ear must be ignored and attention must be directed to the other. Neural injuries/dysfunctions resulting from strokes in the SG compromise these auditory skills, and consequently, healthy auditory processing of acoustic information in the brain.

Another important issue in the dichotic listening task is that when an individual is asked to respond to different verbal stimuli presented simultaneously to each ear, the stimuli are processed predominantly by the contralateral auditory pathway owing to the suppression of its ipsilateral counterpart.^28^^,^^29^

As the hemispheric specialization of language occurs in the left hemisphere, in most cases, the right ear usually performs better, a phenomenon known as right ear advantage.^15^ In this study, when analyzing only individuals with injuries in the right or left hemisphere, the authors found that, as expected, the majority performed worse in the ear contralateral to the lesion owing to the nature of the test.^40^ In addition, the authors found abnormal ipsilateral results, similar to those obtained in other studies.^30^^,^^41^ These different results could be due to the extent of the injuries, and hemispheric reorganization may have occurred.^30^^,^^41^^,^^42^

SG performed worse in the PPS test in both the labeling and humming modes. This result showed that the SG was inefficient at perceiving, associating, and interpreting the non-verbal patterns of the message received, such as rhythm and intonation, which are most often processed by the right hemisphere, affecting comprehension with regard to phoneme voicing and syllable order.^43^, ^44^, ^45^, ^46^

The present results corroborate those of previous studies, demonstrating that individuals with brain injury perform worse on the PPS than normal individuals, regardless of the affected hemisphere or injury location.^30^^,^^47^ These findings demonstrate that the PPS is a highly sensitive test for brain damage since the required skills involve several brain areas from both the hemispheres, such as the primary auditory cortex, auditory association areas, and language-related regions (temporoparietal) when a verbal response is needed.^48^

The activation of these different regions of the brain is associated with several neural processes required to perform this test. Recognizing an acoustic pattern and intonation, presumably, has been predominantly associated with the right hemisphere (humming mode). When the task requires labeling, in addition to the right hemisphere, the corpus callosum and language-related regions in the left hemisphere are activated.^47^^,^^48^

Electrophysiological tests contribute to behavioral assessments of auditory processing because they objectively check the functional and structural integrity of the auditory pathway in the brain.^23^^,^^49^

In this study, the P300 was used because this potential is associated with cognitive skills related to the auditory system and reflects activity in the auditory cortex in terms of attention, discrimination, integration, and memory.^48^^,^^49^

The results obtained in the SG showed an increased latency compared to those in the CG. Thus, the authors can infer that the central auditory nervous system takes longer to process auditory information in children and adolescents with brain injuries. The damage caused by the injury itself likely impairs the normal functioning of neural pathways, causing auditory and cognitive function disorders. Some studies on brain disorders have demonstrated that this neural slowness is a result of injury.^50^^,^^51^ This type of disorder has also been described in studies on children with language development disorders,^52^ learning disorders,^53^ stuttering,^54^ and aging.^55^

The P300 amplitude has been widely questioned in the literature owing to its high variability^56^^,^^57^ and attentional influence. However, it is noteworthy that SG had higher amplitude values than CG.

Vaughan and Kurtzberg studied children and adolescents with functional neurological disorders and found increased amplitudes in this population compared to their healthy peers. The authors hypothesized that these participants would exhibit cortical excitation. The baseline state of high excitation could be a pre-condition for generating functional neurological symptoms in these individuals, justifying their higher potential amplitude when compared to controls, which is a plausible hypothesis because stroke may cause interhemispheric imbalance.^58^ Another hypothesis that the authors proposed was the possible compensation for attentional levels in the SG when performing the discrimination task required in the P300 potential.

In this study, the small sample size represented one of the limitations and reinforced the series of diseases presented in the literature, since the authors identified only 35 children or adolescents diagnosed with stroke over a period of 15 years (2003 to 2018), of which 24 were eligible for the analyses. Another limiting factor for this investigation was the scarcity of audiometric studies involving children with stroke-related neurological sequelae caused by stroke. Therefore, the authors emphasize the importance of future studies that address these issues in greater detail.

## Conclusion

This study showed that children and adolescents with stroke performed worse in electrophysiological and behavioral tests of auditory processing assessed using auditory evoked potentials. These data reinforce the hypothesis that stroke-related lesions compromise the neural mechanisms underlying auditory processing.

## Data availability statement

The authors declare that all data are available to reviewers of the journal.

## Authors' contributions

Andréia Rodrigues Parnoff Stadulni: Offered assistance for the idealization of the research, carried out data collection, analysis and writing of the manuscript.

Pricila Sleifer: Contributed with methodological guidance.

Amanda Zanatta Berticelli: Assisted in the process of collecting and classifying research data.

Rudimar Riesgo: Pediatric neurologist responsible for the evaluations of the participants.

Carolina Nunes Rocha Muniz: Performed the statistical analyses.

Eliane Schochat: Responsible for designing the research topic, revising the manuscript, and providing methodological guidance.

## Funding

CAPES/USP.

## Declaration of Competing Interest

The authors declare no conflicts of interest.
